# Chicago Medical Society—Regular Meeting January 24, 1862

**Published:** 1862-01

**Authors:** 


### Proceedings of Societies.

### CHICAGO MEDICAL SOCIETY—REGULAR MEETING, JANUARY 24TH, 1862.

### DISCUSSION ON THE NATURE AND TREATMENT OF CONSTIPATION.

The Society was called to order by the Vice President, Dr. WICKERSHAM. The reading of the minutes and the report of the Sanitary Committee, were deferred until the next meeting. Dr. HOLMES reported two cases, in which bad symptoms appeared from the inhalation of chloroform, but neither of them proved fatal. He also reported a case of extirpation of the eye-ball, on account of a protracted and very painful inflammation, resulting from mechanical injury to the eye. Dr. D. D. WAITE, who had been appointed to read a paper at this meeting, was granted another week of time.

The subject for discussion, namely, *Constipation and its Treatment*, was then taken up:—

Dr. G. PAOLI opened the discussion by stating that habitual constipation was a condition of the alimentary canal of frequent occurrence, and almost always difficult to relieve permanently. He regarded want of action in the muscular coat of the intestine as the essential cause of the constipation; and consequently, that active hydragogue cathartics, such as calomel, castor oil, salts, etc., were not only useless, but often positively injurious. He thought the proper remedies were such as would increase the contractility of the involuntary muscular structures, and thereby render more efficient the natural peristaltic motion of the intestines. His favorite remedy is strychnia dissolved in water, in the proportion of one grain to the ounce, of which he recommends from five to ten drops to be given twice a day.

Dr. HATCH stated that he had met with cases of habitual constipation in this city much less frequently, than in the community in which he formerly practiced in the State of Vermont. He had met with one species of constipation, frequently in children, and sometimes in adults, accompanied by profuse vomiting of a thin, acrid and sour fluid. It was often accompanied also, by soreness of the mouth. He had pretty uniformly succeeded in relieving this state of the alimentary canal, by dissolving 16 grs. of argenti nitras, and 24 grs. of iodide of potassa, in 4 oz. of water, and giving a teaspoonful after each meal until the vomiting ceased to recur. Then he substituted the use of a cold infusion of hydrastis canadensis until the regularity of the bowels was established. This same treatment he had found more effectual in relieving the *nursing sore mouth*, than any other that he had tried.

Dr. Waite remarked that constipation was a relative term. That many persons passed more than 24 hours habitually without alvine evacuations, and yet appeared to be neither costive nor injured in health. He also alluded to those cases on record, of persons who had passed weeks and months without any intestinal discharges. As a general rule he would regard a patient as constipated, whose evacuations do not occur as often as once in two days. He thinks that in 19 cases out of 20, constipation arises from erroneous habits, refinements in cooking, etc.;—agrees with Liebig, that to ensure regular fœcal evacuations, the food must be such as to leave sufficient fœcal residue to stimulate the intestines to action. In the treatment of constipation he relies mostly upon the correction of errors in habits, diet, and quality of food, and rarely resorts to medicines. He advises patients to exercise actively, and to live largely upon vegetables and coarse bread, especially that made of unbolted flour. If medicine becomes absolutely necessary, he uses a pill composed of blue mass 2 grs., ipecac 2 grs., and aloes 1 gr., to be given every night until a healthy action is established; but never to produce a cathartic effect. He thinks aloes has a specific action on the rectum, and is consequently contra-indicated when hemorrhoids exist.

Dr. Peterson thought constipation not a disease, but only a symptom. Hence we must always inquire after the cause, and apply the remedies to its removal.—If caused by want of action in the muscular coat of the intestines, he would use strychnine or small doses of rhubarb root at bed time. He thinks habitual constipation less frequent here than in Europe.

Dr. Hinkley, being present as a visitor, remarked that he was in the habit of giving black mustard-seed in doses of a teaspoonful daily, wlth very satisfactory results. He thinks hemorrhoids exist in a large proportion of the cases of constipation.

Dr. Davis, stated that he regarded habitual constipation as a symptom, in the same sense as dropsy or dyspnœa. He regarded the cases occurring in practice as capable of being grouped into three classes, viz: those arising from deficient contractility in the muscular coat of the intestines—those arising from deficient secretory action in the follicles and glands of the intestinal mucous membrane—and those induced by deficient secretion from the liver. The first may be produced by any and all those causes that debilitate the nervous and muscular structures, such as sedentary habits; confined and impure air; insufficient supply of air from confinement of the chest, by bad modes of dress; refusal to attend to the calls for defecation when they exist, etc. The second may arise from any of those causes that are capable of diminishing secretory action, and often exists in connection with the first. The third may arise from any pathological state which prevents the bile from passing into the duodenum. Absence of bile, by no means always induces constipation, but such is generally the result. Of course, in treating constipation, due attention should be given to a removal of all such causes as may have contributed to produce it. The patient should practice going to stool at a given time each day; all errors in dress, diet, and exercise should be corrected. Among the best remedies to restore a healthy peristaltic action, he thinks is the following combination:—

℞—Extract. Hyoscyamus, - grs.xxx.
Sulph. Ferri, - - - grs.xxx.
Pulv. Aloe, - - - - grs.x.
Extract. Nux Vom., - grs.x.

Mix and divide into 30 pills—one of which may be given before each meal. In the constipation of chlorotic females and anæmic persons generally, he advises the following:—

℞—Citrate of Iron, - - - - ʒj.

Strychnine, - - - - - grj.

Mix and divide into 30 pills—one of which is to be taken before each meal.

When the constipation arises principally from want of proper secretory action, he has used with much advantage, before each meal, small doses of a solution of sulphate of magnesia acidulated with aromatic sulphuric acid.

Some further remarks were made by Drs. Peterson, Waite, and others.

Dr. W. H. Byford was appointed to read a paper at the meeting, two weeks from this date.

*Hydrocephalus*, was chosen as the subject of discussion for the next meeting—and Drs. Waite and Peterson to lead the discussion.

